# Evaluation of PD-L1 Expression and Systemic Inflammatory Blood Cell Ratios as Prognosticators in Advanced Lung, Breast and Head and Neck Cancers

**DOI:** 10.3390/cancers18111819

**Published:** 2026-06-01

**Authors:** Taoufik Nedjadi, Sultanah AlBoraie, Wardah Alghamdi, Raghad Alkharouby, Lama Almuraee, Dalal Malibari, Alaa Samkari, Samera Alosairi, Rawiah Alsiary, Mohamed Bilal Nedjadi, Majed Ramadan, Mohamed Eldigire Ahmed

**Affiliations:** 1 King Abdullah International Medical Research Centre, King Saud Bin Abdulaziz University for Health Sciences, Ministry of National Guard-Health Affairs, Jeddah 21423, Saudi Arabia; 2 Department of Medicine, College of Medicine, King Saud Bin Abdulaziz University for Health Sciences, Jeddah 21423, Saudi Arabia; 3 Department of Pathology and Laboratory Medicine, Ministry of National Guard-Health Affairs, Jeddah 22384, Saudi Arabia; 4 Department of Medicine, University College London, London WC1E 6BT, UK; 5 College of Science and Health Professions, King Saud Bin Abdulaziz University for Health Sciences, Jeddah 21423, Saudi Arabia

**Keywords:** PD-L1, biomarker, cancers, prognosis, platelet-to-lymphocyte ratio, bioinformatics analysis

## Abstract

Introduction: Immunotherapy with immune checkpoint inhibitors (ICIs) targeting the PD-1/PD-L1 axis has become an established treatment strategy across various malignancies. In this context, PD-L1 expression plays a key role in the clinical management of cancer patients. However, the prognostic significance of PD-L1 expression remains inconsistent across different cancer types. This study aimed to evaluate the prevalence of PD-L1 expression in three cancer types and to examine its association with key clinicopathological parameters, systemic inflammatory markers and patient outcomes. Methods: A retrospective cohort of 141 patients with lung, breast and head and neck cancers diagnosed between 2016 and 2021 was analysed. PD-L1 expression was analysed by immunohistochemistry using clone 22C3 and the scoring system for positivity was determined according to standard clinical scoring practices for each tumour type. Systemic inflammatory markers, including the platelet-to-lymphocyte ratio (PLR), were calculated from pre-treatment blood counts. Correlation and survival analyses were performed to evaluate the associations between PD-L1 expression levels, clinicopathological characteristics and prognosis. Bioinformatics analyses using GeneMania, STRING and TIMER (v.3) databases were conducted to explore functional enrichment, protein–protein interactions and immune-cell infiltration associated with PD-L1 expression. Results: PD-L1 positivity (≥1%) was observed in 56.75% of lung cancer cases, 44% of breast cancer cases and 92.85% of head and neck cancer cases. PD-L1 expression alone was not significantly associated with overall survival across the cancer cohorts. However, it was significantly associated with PLR in the lung cancer cohort (*p* = 0.036). Notably, low PLR was associated with improved survival in both lung cancer (*p* = 0.007) and breast cancer (*p* = 0.031). Bioinformatics analysis identified several PD-L1-interacting genes, including PD-1, CTLA4 and PTPN and demonstrated strong positive correlations between PD-L1 expression and the infiltration of CD8+ T cells, neutrophils and dendritic cells across the analysed malignancies. Conclusions: Despite its high expression in advanced solid tumours, PD-L1 showed limited prognostic value in our cancer cohort. In contrast, systemic inflammatory markers particularly PLR, emerged as a potential indicator of clinical outcome. Our data suggest that integrating PD-L1 status with systemic inflammatory markers may improve outcome prediction and help inform therapeutic decision-making in patients with advanced malignancies.

## Simple Summary

Immune checkpoint inhibitors (ICIs) targeting the PD-1/PD-L1 axis have revolutionised the landscape of cancer treatment. PD-L1 is an established biomarker used to guide immunotherapy decisions in several types of cancer. However, its value in predicting patient survival remains inconsistent and may vary between cancer types. In this study, we evaluated PD-L1 expression in patients with advanced lung, breast, and head and neck cancers and examined its relationship with clinicopathological features, overall survival and systemic inflammatory blood markers. Although PD-L1 expression was common across the three cancer groups, it was not significantly associated with overall survival in any of them. In contrast, the platelet-to-lymphocyte ratio, a simple blood-based inflammatory marker, showed a significant association with PD-L1 expression in lung cancer and emerged as an important prognosticator, particularly in lung and breast cancers. Complementary bioinformatics analysis revealed that PD-L1 is linked to immunoregulatory gene network and positively correlated with CD8+ T cell, neutrophil and dendritic cell infiltration across all three cancer types. Collectively, these findings highlight the limited standalone prognostic utility of PD-L1 in advanced solid tumours and suggest that integrating tumour biomarkers with systemic inflammatory markers may offer a more comprehensive and clinically useful approach for predicting outcomes in patients with advanced cancers.

## 1. Introduction

Cancer remains a major global health challenge and is a leading cause of morbidity and mortality worldwide [1]. According to the International Agency for Research on Cancer (IARC) reports, approximately 20 million new cancer cases and nearly 10 million cancer-related deaths were reported in 2022 [2]. Although lung cancer screening has significantly improved early detection, many patients still present with advanced disease at diagnosis, which directly affects clinical management and survival [3,4]. Similarly, breast cancer screening has markedly improved early detection worldwide including in Saudi Arabia in particular with a 5-year survival exceeding 80% of breast cancer patients across most regions and exceeding 90% in the United States [5,6]. Despite these advances, a substantial proportion of patients continue to be diagnosed with locally advanced or metastatic disease, for which prognosis remains poor [7,8]. For most patients with locally advanced or metastatic disease chemotherapy remains a mainstay of treatment. However, its severe side effects and the emergence of drug resistance highlight the growing need for alternative therapeutic strategies [9]. Immunotherapy has emerged as a promising approach to cancer treatment providing unprecedented efficacy and improved therapeutic comfort, and has rapidly been adopted as a preferred treatment option for patients with various malignancies [10,11]. Immune checkpoint inhibitors (ICIs) exploit interactions between immune cells and cancer cells to disrupt the inhibitory signals used by tumours to evade immune detection [12]. Accumulating evidence showed that ICIs enhance the cytotoxic efficiency of CD8+ and reverse the immunosuppressive environment within the immune-rich tumour microenvironment (TME). Therefore, the immune-cell composition of the TME plays an important role in the success of ICI therapy [13,14,15]. Targeting Programmed Cell Death Protein-1 (PD-1) and its ligand Programmed Cell Death Ligand-1 (PD-L1) using FDA-approved agents has become a standard first-line treatment for locally advanced and metastatic tumours [16,17,18]. Assessing PD-L1 expression is therefore important in clinical decision-making, as it helps identify patients who are more likely to benefit from ICI therapy. Recent studies have demonstrated the prognostic significance of PD-L1 expression and its predictive value as a biomarker associated with improved outcomes after anti-PD-L1 therapy [19,20]. The KEYNOTE-024 study indicated that PD-L1 expression is a prerequisite for improved OS and PFS with Pembrolizumab in NSCLC [10]. However, high PD-L1/PD-1 co-expression has been associated with significantly lower survival rates, marking this combination as a poor prognostic indicator in breast cancer and oral squamous cell carcinoma patients [21,22]. In advanced NSCLC, PD-L1 positivity has been associated with larger tumour size, increased rates of neural and vascular invasion, lymph node metastasis and worse prognosis in patients with advanced-stage disease [23]. These inconsistent findings may be partly explained by the recent evidence suggesting that changes in the peripheral blood content, including exosomes and ctDNA, may influence cancer progression and therapeutic response. Emerging evidence also suggests that systemic inflammatory markers such as the neutrophils-to-lymphocyte ratio (NLR) and platelet-to-lymphocyte ratio (PLR) may be implicated in the immunotherapy response and could serve as potential indicators for monitoring disease progression and patients’ outcomes across multiple malignancies [24,25,26]. Based on these findings, we conducted a retrospective study to evaluate the prognostic significance of PD-L1 expression in cancer patients diagnosed with advanced diseases including lung, breast and head and neck cancers. Furthermore, we investigated the association between PD-L1 expression and various clinical and pathological parameters, including the relationship with systemic inflammatory indices such as PLR and NLR. In addition, we aimed to provide a broader understanding of the role of PD-L1 within the TME by integrating bioinformatics analyses of immune infiltration, gene ontology enrichment and protein–protein interactions.

## 2. Materials and Methods

### 2.1. Sample and Data Collection

The current study was conducted under a retrospective cohort design and included 141 patients diagnosed with various advanced malignancies at King Abdulaziz Medical City, NGHA- Jeddah, between March 2016 and April 2021. The study population includes patients diagnosed with lung cancer, breast cancer and head and neck cancer. This study was approved by the institutional ethics committee (IRB N. 0342/23) and was conducted in accordance with the Declaration of Helsinki. The Institutional Review Board waived the requirement for informed consent. Clinical and pathological characteristics were collected retrospectively through review electronic medical records using the BestCare data management system.

### 2.2. Immunohistochemistry Staining and Scoring

Samples from patients diagnosed with three different cancer types were tested for PD-L1 expression status as part of their clinical management of these patients. PD-L1 staining was performed on formalin-fixed paraffin-embedded tissue sections at Targos Molecular Pathology GmbH (Kassel, Germany) using the FDA-approved pharmDx assay (Agilent Technologies, Santa Clara, CA, USA) with the PD-L1 22C3 clone antibody. Stained slides were evaluated and scored by experienced clinicians from Targos Molecular Pathology GMBH, and results were disseminated in standardised electronic reports.

The cut-off for PD-L1 staining positivity, defined as protein expression on the cell membrane of cancer cells, depends on the cancer type. For instance, breast and head and neck cancers were assessed using the Combined Positive Scoring (CPS) system, whereas lung cancer cases were evaluated using the Tumour Proportion Scoring (TPS) system. Staining positivity cut-off follows the scoring guidelines used by the laboratory. Briefly, for the TPS system, the pathologist assesses PD-L1 positivity by counting viable tumour cells demonstrating complete or partial membrane staining. Positivity in the tumour-associated immune cells such as macrophages was excluded from evaluation. The tumour proportion score (TPS) was determined as follows: TPS (%) = (Positive PD-L1 tumour cells/Total number of tumour cells) × 100. All tumours with TPS ≥ 1% were considered PD-L1 positive. For the CPS, PD-L1 expression in both tumour cells and associated immune cells was calculated using the following formula: CPS (%) = Number of PD-L1-positive cells (tumour cells and immune cells)/total number of viable tumour cells × 100.

**Figure 1:**
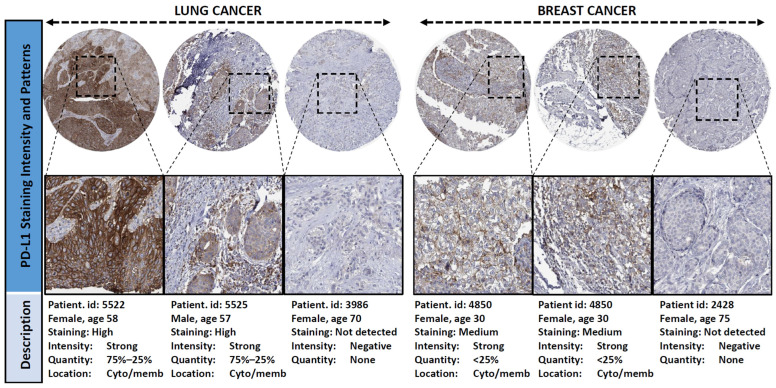
Representative immuno-staining patterns of PD-L1 expression in the tumour cells and/or stroma cells of lung and breast cancers extrapolated from the Human Protein Atlas Database (https://www.proteinatlas.org. Accessed on the 17 May 2026).

Because immunohistochemical (IHC) images from the patient cohort were not available, images from the Human Protein Atlas (HPA) database were used as representative examples of positive and negative PD-L1 expression patterns in lung and breast cancers (https://www.proteinatlas.org/, accessed on the 17 May 2026). The HPA represents a comprehensive publicly available database that provides high-resolution protein expression data covering a wide range of cancer types. As shown in Figure 1, PD-L1 expression can be found in the tumour cells exhibiting both cytoplasmic and membranous positivity and/or in cells associated with the microenvironment.

### 2.3. PD-L1 and Immune-Cell Ratios

Additional information related to the immune-cell laboratory counts was collected prior to patients receiving any treatment. The CBC counts were retrieved from patients’ medical records through the BestCare system and included neutrophil, monocyte, lymphocyte and platelet counts. The immune-cell ratios were calculated as previously described [27], briefly:

- Neutrophil-to-lymphocyte ratio (NLR): the result of absolute neutrophil count divided by absolute lymphocyte count.
- Platelet-to-lymphocyte ratio (PLR): the result of absolute platelet count divided by absolute lymphocyte count.
- Lymphocyte-to-monocyte ratio (LMR): the result of absolute lymphocyte count divided by absolute monocyte count.

Kaplan–Meier survival curves were used to assess the relationship between PLR ratio and overall survival. Receiver operating characteristic (ROC) curve analysis was undertaken to determine the cut-off values defining high and low PLR across the cancer cohorts.

### 2.4. Functional Enrichment and PPI Analysis

Next, we sought to perform functional enrichment analysis using the GeneMania database to identify neighbouring and target gene networks associated with PD-L1 (https://genemania.org/search/homo-sapiens/PD-L1. Accessed on the 23 March 2026). Gene Set Enrichment Analysis (GSEA) was then performed on the PD-L1-interacting network to identify significantly associated biological processes and cellular components. The STRING platform was then used for protein–protein interaction analysis (https://string-db.org. Accessed on the 20 March 2026). In order to focus the network list to the strongest and most reliable interactions we adopted a strict confidence cut-off (>0.900) and limited the first shell to no more than 20 interactions. TIMER (v.3) was used to evaluate immune-cell infiltration.

### 2.5. Statistical Analysis

Data analysis was carried out using JMP Pro statistical software version 18.0. Frequencies and percentages were used to summarise categorical variables included in the study, while means and standard deviations (SDs) were calculated for the continuous variables. Association between PD-L1 expression and study variables were evaluated using the Chi-square test and Fisher’s exact test, as appropriate. An unpaired *t*-test was used to examine the significant differences in blood parameters according to PD-L1 status. Kaplan–Meier curves were used to illustrate the overall survival (OS) by the PD-L1 status. Predictors of OS were identified by a Cox-regression model adjusted for patient age and gender. A *p*-value of less than or equal to 0.05 was set to be a statistically significant result.

## 3. Results

### 3.1. Baseline Characteristics of the Cohort

Between March 2016 and April 2021, a total of 141 patients were tested for PD-L1 and included in this study of whom 62 were females (44%) and were 79 males (56%). Patients were diagnosed with lung cancer (52.5%), breast cancer (17.7%) or head and neck cancer (29.8%) and were treated at King Abdulaziz Medical City—Jeddah. The lung cancer cohort included 74 patients with a mean age of 62.4 years; 57 patients (77.0%) were male. The median duration from diagnosis to last follow-up was 11.0 months (IQR 5.0–16.0), the median months of survival was 6.5 months (IQR 2.8–10.2) and 45.4% of patients were smokers. The baseline characteristics of the analysed cohort are summarised in Table 1. The most prevalent comorbidities were diabetes (56.8%), hypertension (47.3%) and dyslipidaemia (21.6%). The majority of these patients were diagnosed with advanced-stage diseases (stage 3/4). The cohort was further characterised according to the most common disease-specific mutations or biomarker expression patterns. Lung cancer patients harbour mutations of TTF1 (18.9%), CK7 (18.9%) and TP53 (17.6%) genes. The breast cancer cohort included 25 patients, with a mean age of 57.5 years. Among these patients, 60% were alive at last follow-up, the median duration from diagnosis to last follow-up was 10.0 months (IQR 7.0–45.0) and the median survival was 22.5 months (IQR 12.3–40.3). Hypertension (48.0%), diabetes (28.0%) and dyslipidaemia (28.0%) were the most prevalent comorbidities. PR positivity was the predominant biomarker status identified (52.0%), ER positivity and triple-negative cases represented 28.0% and 4.0% of the analysed cohort, respectively. The head and neck group exhibited mutation of the P63 and CK5/6 genes (Table 2). The head and neck cancer cohort included 42 patients of whom 52.4% were male, with a mean age of 63.9 years. The median duration from diagnosis to last follow-up was 17.0 (IQR 12.0–36.0) months and the median survival was 17.0 months (IQR 4.5–37.5). Hypertension (42.8%), diabetes (40.5%) and IHD (14.3%) were the most common comorbidities. Genetic testing revealed P63 (4.8%), CK5/6 (4.8%) and EML4-ALK (2.4%) as the most common reported mutations.

### 3.2. PD-L1 Expression Across Malignancies

The cut-off point for PD-L1 expression was largely based on the antibody clone tested and the type of tumour analysed. In general, positivity was determined at ≥1% positive tumour cells. In the lung cancer cohort, positive PD-L1 expression was reported in 42 of 74 (56.75%) patients whereas in the breast cancer and head and neck cancer cohorts, PD-L1 positivity was identified in 44% (11/25) and 92.85% (39/42) of patients, respectively. Representative images of positive and negative PD-L1 expression patterns in lung and breast cancers, assessed by immunohistochemistry were adopted from the Human Protein Atlas (HPA) database (https://www.proteinatlas.org/. Accessed on the 17 May 2026). The HPA represents a comprehensive publicly available database containing high-resolution protein expression images covering several cancer types [28]. As shown in Figure 1, PD-L1 expression can be observed in the tumour cells exhibiting both cytoplasmic and membranous positivity and/or in cells associated with the tumour microenvironment.

### 3.3. Association Between PD-L1 Expression and Clinicopathological Characteristics

Statistical analysis was carried out to assess associations between PD-L1 expression status (positive vs. negative) and selected clinical and pathological variables. Our data indicated that PD-L1 expression did not correlate with any of the tested variables in the lung cancer cohort (Table 3). In the breast cancer cohort, PD-L1 expression was significantly associated with disease recurrence (*p* = 0.049). Patients with high PD-L1 expression were also more likely to receive immunotherapy in both the lung cancer (*p* = 0.05) and breast cancer (*p* = 0.014) cohort.

### 3.4. Association Between PD-L1 Expression and Survival

**Figure 2:**
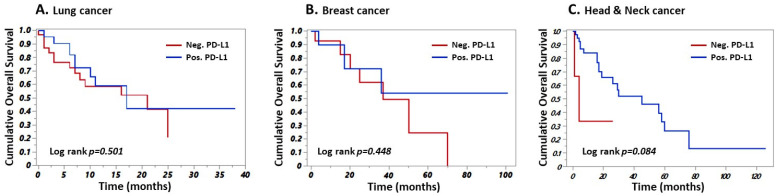
Impact of PD-L1 expression on patient’s survival in lung cancer (**A**), breast cancer (**B**) and head and neck cancer (**C**). Kaplan–Meier survival curves indicated no significant association between PD-L1 expression and overall survival in any cohort.

Then, we sought to investigate the prognostic value of PD-L1 expression in all cancer sub-groups (Figure 2). Our data indicated that PD-L1 expression had no prognostic value in lung cancer patients (log-rank *p* = 0.501). Similar findings were observed in breast cancer (log-rank *p* = 0.448) and head and neck cancer (log-rank *p* = 0.084).

### 3.5. PD-L1 and Immune-Cell Ratios

**Figure 3:**
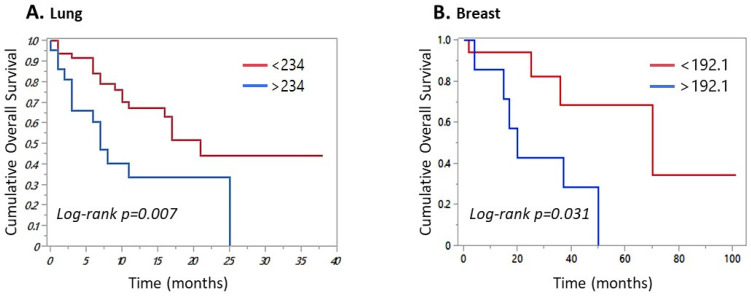
Overall survival analysis based on PLR ratio. (**A**) lung cancer and (**B**) breast cancer. Kaplan–Meier curves indicated that the high expression level of the PLR ratio is correlated with poor overall survival in lung cancer patients compared to patients with a low PLR ratio (log-rank *p* = 0.007).

**Figure 4:**
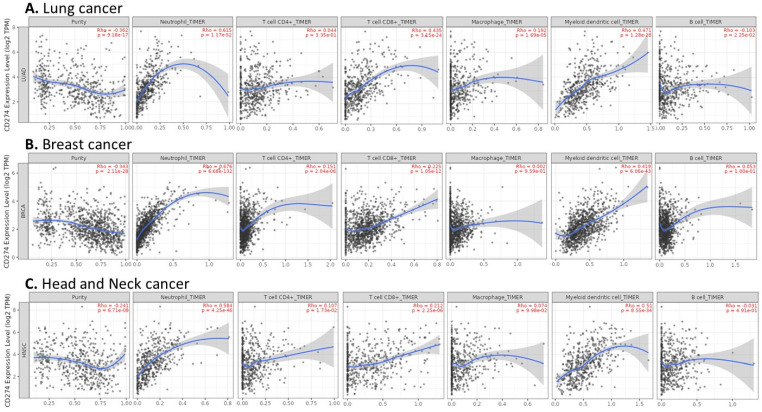
Analysis of immune-cell infiltration in cancer cohorts. Correlations between PD-L1 expression and the abundance level of six immune-cell populations. The data demonstrated significant associations between PD-L1 expression and B cells, CD8^+^ T cells, CD4^+^ T cells, macrophages, neutrophils and dendritic cell infiltration in lung cancer (**A**), breast cancer (**B**) and head and neck cancer (**C**) as revealed by the TIMER (v.3) database (https://compbio.cn/timer3/, accessed on 13 April 2026).

Since the ratios of inflammatory markers are associated with immunotherapy treatment efficacy, we conducted an in-depth analysis to further explore the role of PD-L1 expression in cancer. We therefore assessed the relationship between PD-L1 expression and immune-cell ratios, including LNR, PLR and LMR across the three cohorts. In the lung cancer cohort, our data demonstrated a significant association between PD-L1 expression and PLR ratio (*p* = 0.036), whereas no significant association was observed with LNR or LMR (Table 4A). PLR was not associated with PD-L1 expression in breast or head and neck cancers (Table 4B,C). ROC curve analysis identified PLR threshold values of 234.5, 135.7 and 192.1, as the optimal cut-offs for the best sensitivity and specificity in lung, head and neck and breast cancers, respectively. In lung cancer, PLR was found to be associated with patients’ outcome as patients with low PLR exhibited significantly better overall survival than those with high PLR (log-rank *p* = 0.007) compared with the high PLR group (Figure 3A). Similarly, breast cancer patients with low PLR ratio had better outcomes (log-rank *p* = 0.031) (Figure 3B). However, the PLR ratio did not show any prognostic value in head and neck cancer with a log-rank *p* = 0.52. To delve deeper into the relationship between PD-L1 expression level and immune cells, we used the TCGA data and the TIMER (v.3) program to investigate associations between PD-L1 expression and immune-cell infiltration in lung, breast and head and neck cancers. Our data revealed a significant positive correlation between PD-L1 expression and neutrophils, CD8^+^ T cells and dendritic cells in all cancer types (Figure 4A–C). CD4^+^ T cells were positively correlated with PD-L1 expression only in breast and head and neck cancers. A positive association between macrophages and PD-L1 expression was found in the lung cancer cohort only. In the lung cancer cohort, an inverse relationship was observed between PD-L1 expression and B-cell infiltration (Figure 4A).

### 3.6. Protein Network and Functional Enrichment Analysis

**Figure 5:**
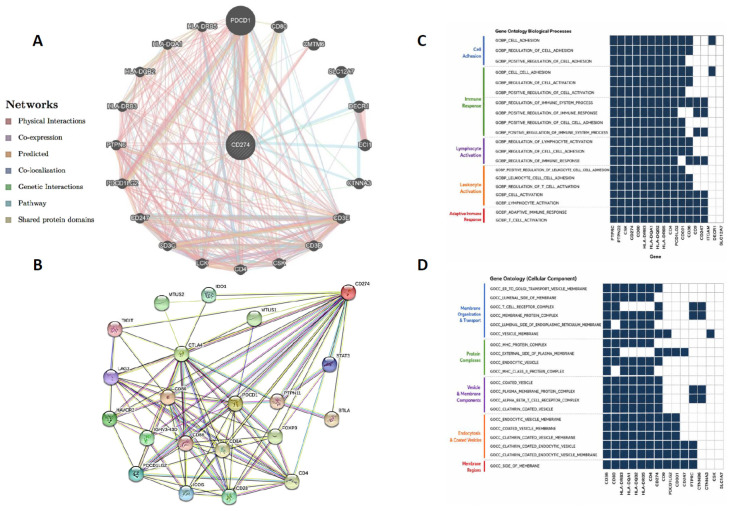
Functional enrichment analysis of the PD-L1 network. (**A**). Construction of PD-L1-interacting gene network generated using the GeneMania database. (**B**) Protein–protein interaction network of PD-L1 showing the top 20 PD-L1-interacting proteins. The figure was generated using the online STRING database with the highest confidence score (>0.9). (**C**). GSEA analysis depicting the most significant biological functions of PD-L1-interacting genes (**D**). Chart demonstrating the most common cellular components in GO analysis.

We then used the GeneMania database to determine the full interacting gene network of the PD-L1 gene (*CD274*). The list of interacting genes included the *PDCD1* gene, which encodes programmed cell death-1 (PD-1), as well as *CD80*, *CD4*, *CD3*, *CMTM6*, *SLC12A7*, *DECR1*, *ECI1*, *CTNNA3* and other genes. These genes shared either physical interactions, co-expression, co-localisation, pathway involvement or shared protein domains (Figure 5A). Furthermore, protein–protein interaction (PPI) analysis using the STRING database further indicated that PD-L1 interacts with several immune checkpoint proteins including PD-1, PD-L2, TIGIT, CTLA4, LAG3, CD4, CD8, CD28, CD80, CD86, immune-related antigens such as PTPN11, BTLA and HAVCR2, in addition to transcription factors such as STAT3 and FOXP3 (Figure 5B). Functional enrichment analysis of PD-L1-interacting genes using the GSEA database showed that these genes are mainly involved in many biological processes related mainly to immune-response regulation, lymphocyte activation and cellular adhesion (Figure 5C). At the level of cellular localisation (GO:CC), the interacting proteins were enriched in membranes of either plasma cell, Golgi vesicles, endoplasmic reticulum and clathrin-coated vesicles (Figure 5D).

## 4. Discussion

Cancer immunotherapy with immune checkpoint inhibitors (ICIs) has revolutionized cancer treatment strategies and has provided an unprecedented breakthrough in the management of multiple malignancies, particularly locally advanced and metastatic disease [29,30]. FDA-approved drugs targeting PD-1 such as Pembrolizumab, Nivolumab and Cemiplimab and PD-L1 such as Atezolizumab, Avelumab and Durvalumab have been approved as first-line therapies for several solid and haematological malignancies and have been associated with significant treatment responses and improved patient outcomes [10,11,31]. However, the limited efficacy of ICIs poses a major clinical challenge as only 20–40% of cancer patients achieve a sustained therapeutic response [32]. These antibodies modify the anti-tumour immunity by deactivating the inhibitory signals generated through the interaction between PD-1 and its ligands PD-L1 and PD-L2. This interaction weakens the cytotoxic T-cell activity, promotes a condition of immune tolerance and exhaustion and creates an immunosuppressive microenvironment that supports tumour growth. PD-L1 plays an important role in immune evading, a complex phenomenon involving crosstalk between tumour cells, the TME and immune cell, mediated through a plethora of cytokines and chemokines that enable uncontrolled tumour growth and progression [33]. Targeting the PD-1/PD-L1 axis therefore represents a prominent and effective strategy in cancer therapy strategy, especially in patients with advanced-stage and metastatic disease [34,35]. It has been shown that PD-L1 expression is upregulated in a spectrum of solid and liquid malignancies underscoring its significance in selecting the most appropriate immunotherapeutic agents [36,37]. Accordingly, IHC testing for PD-L1 has become a clinical standard and is currently used as a diagnostic biomarker and an important tool in the clinical management of multiple tumours. Additionally, PD-L1 expression holds a prognostic special value particularly in lung cancer where increased PD-L1 expression has been significantly associated with disease aggressiveness and poor outcomes and has been approved as a predictive biomarker for anti-PD-L1 therapy [38,39].

In our study, we retrospectively investigated the expression of PD-L1 in patients with lung, breast and head and neck cancers, most of whom were diagnosed with advanced diseases and correlated the findings with a set of clinicopathological features. PD-L1 expression demonstrated varied expression patterns across the tested cancer types, with the highest positivity in head and neck cancer (92.85%) followed by lung cancer (56.75%) and breast cancer (44%). These rates are comparable with published data from Li et al. (2022) who reported that nearly 50% of the lung cancer patients tested with clone 22C3 exhibited positive PD-L1 expression [40]. In a meta-analysis of PD-L1 expression, Petrelli et al. (2018) revealed that 38% (5066/13,279) of NSCLC cases were PD-L1 positive [41]. In contrast, Liu et al. (2022) reported PD-L1 positivity in only 24.9% of lung cancer cases tested by IHC using clone SP263 from Ventana with a cut-off point of ≥1% [42]. Cross-study variation in PD-L1 expression is widely recognised and depends heavily on the scoring system used (TPS or CPS) and the applied cut-off points. In head and neck cancer, findings from the Keynote-048 clinical trial revealed that 23% of patients were PD-L1 positive using TPS > 50 system, while 43% scored positive using CPS > 20 [43]. In the current study, PD-L1 expression alone did not confer prognostic value across the analysed cancers, despite its established role as a predictive biomarker role for ICI therapy. The prognostic value of PD-L1 expression remains inconclusive when used as a standalone determinant of clinical outcome. For instance, in a pan-cancer study, Wang et al. (2024) reported that high PD-L1 expression was associated with better OS and RFS in breast, bladder, ovarian, sarcoma and kidney cancers, a finding consistent with recent clinical data from Wheatley-Price et al. (2025) [44,45]. Conversely, the same study showed that increased PD-L1 expression was significantly associated with unfavourable outcomes in patients with thyroid, pancreatic, lung, head and neck and cervical cancers [45]. This was further supported by a recent finding by Ma and Gu (2026) who demonstrated that high PD-L1 expression was associated with poor outcome in lung cancer during 5 years of follow-up (*p* = 0.008) [46]. In a meta-analysis covering 17 cohort studies, Blazek et al. (2023) demonstrated that PD-L1 had prognostic value when only expressed on immune cells, whereas tumour-cell PD-L1 expression had no prognostic value [47]. Other investigations have reported no significant survival benefit between PD-L1-positive and PD-L1-negative tumours. For example, Ohkuma et al. (2023) demonstrated that PD-L1 expression was not associated with overall survival or progression-free survival in lung cancer patients [48], which is in line with our findings in the lung and breast cancer cohorts. In head and neck cancer, patients showed a trend toward better outcomes with high PD-L1 expression. A recent investigation by Takahashi et al. (2024) found that 43.3% of patients with positive PD-L1 expression had no significant correlation with survival outcomes, further highlighting the limited prognostic utility of PD-L1 [49]. These discrepancies suggest that PD-L1 represents only a limited facet of tumour biology and lacks sufficient depth as standalone prognostic or predictive biomarker. On the other hand, this inconsistency may partially reflect the complexity of the TME, cancer heterogeneity and the influence of systemic inflammation on PD-L1 dynamics.

A key observation in the current study was the significant association between PD-L1 expression and the prognostic value of the systemic inflammatory marker platelet-to-lymphocyte ratio (PLR), suggesting that the functional dynamics of PD-L1 may depend, at least in part, on its interaction with the systemic inflammatory indices such as platelet–lymphocyte ratio (PLR). This interpretation is supported by recent data highlighting the importance of systemic immune markers in cancer progression and tumour–immune-cell interactions. A recent study by Kim et al. (2024) demonstrated that combining immune checkpoint expression with both PLR and NLR in pre-ICI-treated patients improved the prognostic stratification in head and neck carcinoma patients [50]. The authors showed that patients with high PLR and NLR had poorer treatment response and shorter progression-free survival [50]. Our data extend this concept by demonstrating that lung cancer patients with high PLR and breast cancer patients with high PLR and high NLR had unfavourable outcomes, suggesting a potential mechanistic link between tumour immune evasion and systemic inflammatory responses. Emerging evidence also suggested that PLR may serve as a prognostic marker in metastatic breast cancer patients and as a response biomarker in TNBC subjected to ICI-based therapy [51,52].

The exact underlying mechanisms are not fully understood. However, experimental evidence from preclinical studies implicates platelet activation, immune-cell regulation and suppression of NK cell function, collectively in tumour development and progression [53,54,55]. Platelet-induced cancer-cell growth has been shown to be mediated through the release of a plethora of chemokines and cytokines, including TNF-α and IL-6 [56,57,58]. Several other studies have also indicated a role for platelet-related genes and their association with immune checkpoint expression in cancer prognosis [59,60]. Onagi et al. (2023) revealed that patients with elevated PLR and NLR had increased CD3+CD4+FOXP3+ T cells, suggesting that high PLR may create a suppressive immune environment in TNBC [52]. A recent investigation highlighted the critical mechanism by which platelets regulate infiltration by dendritic cells, T cells and tumour-associated macrophages (TAMs), thereby affecting cancer patient outcomes [61]. Importantly, the integration of bioinformatics analysis in our study provided additional insight into the biological role of PD-L1 expression within the TME. We observed that PD-L1 correlated positively with several tested immune-cell populations, including CD4+ T cells, CD8+ T cells, macrophages, B cells and dendritic cells in lung, breast and head and neck cancers. This is consistent with previous reports in hepatocellular carcinoma and cervical carcinoma showing that PD-L1 is closely associated with immune-cell infiltration [62,63]. However, the presence of immune infiltration does not necessarily translate into an effective anti-tumour response as PD-L1-mediated immune suppression often induces T-cell immune exhaustion and impairs anti-tumour immunity [64]. The association between PD-L1 and immune-cell infiltration may reflect either immune activation or immune suppression, which may explain why some PD-L1-positive tumours do not respond to ICIs, while some PD-L1-negative tumours still derive clinical benefit [65,66]. This topic remains insufficiently studied and warrant further research to elucidate the biological function and clinical utility of PD-L1 and the cellular composition of the TME. Recent TCGA-based studies support holistic data interpretation of immune checkpoint molecules, TME composition and gene-expression networks. In our study, PPI analysis demonstrated that PD-L1 is embedded within complex immunoregulatory pathways and cellular processes, in specific locations, which may determine its function. GO mapping analysis identified pathways and genes T cells with physical, co-localisation or co-expression interactions, including PDCD1, CTLA4, CD-80, TIGIT, LAG-3 and PDCD1LG2, which encodes the PD-L2 protein. These findings suggest that PD-L1 does not act in isolation but forms part of a dynamic immunological orchestra. Several PD-L1-interacting genes have already been associated with inhibition of T-cell function and the formation of an immunosuppressive environment in multiple cancers [67]. Moreover, many of these genes serve as prognostic markers in various malignancies and are being actively investigated as targets for cancer therapy [68,69]. Therefore, an in-depth analysis of the complex interactions between these genes and pathways across different cancer types may help clarify mechanisms of resistance in ICI-treated patients, improve survival and maximise the benefit of combination therapeutic strategies.

## 5. Conclusions

In conclusion, our findings highlight the limitations of relying solely on PD-L1 expression for patient stratification. The results underscore the need for an integrated multidimensional approach that incorporates systemic inflammatory markers, immune-cell infiltration and molecular network analysis of genes and pathways. This perspective is supported by recent advances in TME scoring systems and modelling approaches, which consistently demonstrate the superiority of composite biomarkers over single-parameter models as powerful predictors of immunotherapy response [70,71,72]. Despite these insights, this study has several limitations that warrant consideration, including the small sample size, limited subgroup representation and heterogeneity of the analysed cancer cohorts. Furthermore, the investigation was based on the retrospective data from a single-centre database, which may limit the generalisability and the statistical robustness of the findings. Larger prospective multicentre studies are therefore warranted to validate the prognostic value and clinical utility of PD-L1 expression. Finally, the molecular mechanisms governing the relationship between PD-L1 and immune-cell infiltration and gene signature related to anti-tumour immunity require further comprehensive characterisation through integrated multi-OMICs approaches.

## Figures and Tables

**Table 1:** Baseline characteristics of participants summarising the clinical and pathological features of the cancer cohort.

Variable	Lung Cancer Patients (*n* = 74)	Breast Cancer Patients (*n* = 25)	H&N Cancer Patients (*n* = 42)
Age	62.4 ± 11.2	57.5 ± 13.4	63.9 ± 15.5
Gender			
Female	17 (23.0%)	25 (100%)	20 (47.6%)
Male	57 (77.0%)	-	22 (52.4%)
Smoking status			
Yes	16 (21.6%)	2 (8%)	6 (14.3%)
No	25 (33.8%)	21 (84%)	23 (54.8%)
Ex	14 (18.9%)	0 (0%)	2 (4.7%)
Unknown	19 (25.7%)	2 (8%)	11 (26.2%)
Status			
Live	44 (59.5%)	15 (60)	21 (50)
Died	30 (40.5%)	10 (40)	21 (50)
Tumour Grade			
Well tumour differentiation	4 (5.4%)	0 (0%)	9 (21.4%)
Moderate tumour differentiation	19 (25.7%)	8 (32%)	20 (47.6%)
Poor tumour differentiation	16 (21.6%)	16 (64%)	5 (11.9%)
Undifferentiated	2 (2.7%)	1 (4%)	5 (11.9%)
Unknown	33 (44.6%)	-	3 (7.2%)
Comorbidities			
Diabetes	56.8%	28%	40.5%
Hypertension	47.3%	48%	42.8%
Dyslipidaemia	21.6%	28%	11.9%
Ischemic Heart Disease	8.1%	-	14.3%
Hypothyroidism	2.7%	8%	7.1%
Chronic Kidney Disease	2.7%	12%	7.1%

**Table 2:** List of common mutations and biomarker expression profile across the cancer cohorts. * Denotes biomarker status in cancer rather than gene mutation.

	Mutations/Expression (%)		
Genes	Lung Cancer (%)	Head and Neck Cancer (%)	Breast Cancer (%)
*BRAF*	2.7	-	-
*TTF1*	18.9	-	-
*KRAS*	17.6	-	-
*Napsin A*	12.2	-	-
*EGFR*	12.1	-	-
*P63*	9.5	4.8	-
*EML4-ALK*	8.1	2.4	4
*CK5/6*	5.4	4.8	12
*CK7*	18.9	-	52
*BRCA-1*	-	-	8.1
Ki-76 *	-	-	20
ER+ *	-	-	28
PR+ *	-	-	52
Triple Negative *	-	-	4

**Table 3:** Association analysis between the expression level of PD-L1 and clinicopathological variables.

	Lung Cancer				Head and Neck Cancer				Breast Cancer			
Parameters	*n* = 74	Neg. PD-L1	Pos. PD-L1	*p*-Value	*n* = 42	Neg. PD-L1	Pos. PD-L1	*p*-Value	*n* = 25	Neg. PD-L1	Pos. PD-L1	*p*-Value
LN Metast.	15 (20.3)	6 (18.8)	9 (21.4)	0.776								
Lung Metast.	6 (8.1)	3 (9.4)	3 (7.1)	0.782								
Liver Metast.	7 (9.5)	2 (6.3)	5 (11.9)	0.342								
Bone Metast.	20 (27.0)	7 (21.9)	13 (31.0)	0.384								
Brain Metast.	18 (24.3)	10 (31.3)	8 (19.1)	0.227								
No Mets	23 (31.1)	7 (21.9)	16 (38.1)	0.135								
Curative treat.	17 (37.0)	7 (35.0)	10 (38.5)	0.809	9 (33.3)	1 (50.0)	8 (32.0)	0.897	9 (60.0)	4 (57.1)	5 (62.5)	0.622
Palliative treat.	29 (63.0)	13 (65.0)	16 (61.5)		18 (66.7)	1 (50.0)	17 (68.0)		6 (40.0)	3 (42.9)	3 (37.5)	
Chemotherapy	51 (73.9)	21 (75.0)	30 (73.2)	0.865	30 (76.9)	2 (100)	28 (75.7)	0.587	24 (96.0)	14 (100)	10 (90.9)	0.440
Surg. therapy	12 (17.4)	6 (21.4)	6 (14.6)	0.464	22 (56.4)	1 (50.0)	21 (56.8)	0.688	15 (60.0)	11 (78.6)	4 (36.4)	0.042
Radiotherapy	28 (40.5)	9 (32.1)	19 (46.3)	0.238	30 (76.9)	1 (50.0)	29 (78.4)	0.413	11 (44.0)	6 (42.9)	5 (45.5)	0.607
Targ. therapy	14 (20.3)	6 (21.4)	8 (19.5)	0.846	2 (5.1)	0 (0)	2 (5.4)	0.899	5 (20.0)	5 (100)	0 (0)	0.038
PDL therapy	26 (38.2)	8 (29.6)	18 (43.9)	0.236	15 (39.5)	0 (0)	15 (41.7)	0.359	7 (28.0)	2 (14.3)	5 (45.5)	0.101
Hormone ther.	3 (4.3)	1 (3.6)	2 (4.9)	0.641	1 (2.6)	0 (0)	1 (2.7)	0.948	4 (16.0)	4 (100)	0 (0)	0.079
Recurrence	7 (11.7)	2 (8.3)	5 (13.9)	0.511	15 (41.7)	1 (33.3)	14 (42.4)	0.627	3 (13.0)	0 (0)	3 (100)	0.049
Well diff. tumour	4 (9.7)	2 (12.5)	2 (8.0)	0.995	9 (23.1)	1 (33.3)	8 (22.2)	0.187	0 (0)	-	-	0.658
Mod. dif. tumour	19 (46.3)	7 (43.8)	12 (48.0)		20 (51.3)	2 (66.7)	18 (50.0)		8 (32.0)	5 (35.7)	3 (27.3)	
Poor diff. tumour	16 (39.0)	6 (37.5)	10 (40.0)		5 (12.8)	0 (0)	5 (13.9)		16 (64.0)	9 (64.3)	7 (63.6)	
Undiff. tumour	2 (4.9)	1 (6.3)	1 (4.0)		5 (12.8)	0 (0)	5 (13.9)		1 (4.0)	0 (0)	1 (9.1)	
Immunotherap.	32 (43.2)	10 (31.3)	22 (52.4)	0.050	19 (45.2)	0 (0)	19 (48.7)	0.154	11 (44.0)	3 (21.4)	8 (72.7)	0.014

**Table 4:** Association analysis between PD-L1 expression and immune-cell ratios in (**A**) lung cancer, (**B**) breast cancer and (**C**) head and neck cancer.

(**A**)				
Parameter	*n* = 74	Negative PD-L1	Positive PD-L1	*p*-value
LNR	4.52 ± 0.57	4.69 ± 0.88	4.40 ± 0.76	0.806
PLR	214.8 ± 13.0	242.5 ± 23.6	90.5 ± 14.2	0.036
LMR	3.01 ± 1.99	2.62 ± 1.69	3.29 ± 2.15	0.162
(**B**)				
Parameter	*n* = 25	Negative PD-L1	Positive PD-L1	*p*-value
LNR	2.5 ± 1.7	2.7 ± 1.8	2.3 ± 1.8	0.632
PLR	173.9 ± 21.4	174.7 ± 29.3	173.0 ± 32.7	0.970
LMR	4.2 ± 2.3	4.5 ± 2.1	3.9 ± 2.6	0.540
(**C**)				
Parameter	*n* = 42	Negative PD-L1	Positive PD-L1	*p*-value
LNR	5.9 ± 1.3	4.3 ± 1.0	6.0 ± 1.3	0.775
PLR	235.8 ± 38.6	294.5 ± 28.6	253.8 ± 40.5	0.735
LMR	2.5 ± 1.5	1.6 ± 0.5	2.6 ± 1.5	0.267

## Data Availability

The datasets generated during and analysed during the current study are available from the corresponding authors upon reasonable request.
